# Hydrolytic Modification of SiO_2_ Microspheres with Na_2_SiO_3_ and the Performance of Supported Nano-TiO_2_ Composite Photocatalyst

**DOI:** 10.3390/ma14102553

**Published:** 2021-05-14

**Authors:** Yu Tu, Weihua Ao, Chunhong Wang, Tianyu Ren, Lijuan Zhang, Jiaxin Zhong, Wei Li, Hao Ding

**Affiliations:** 1 Beijing Key Laboratory of Materials Utilization of Nonmetallic Minerals and Solid Wastes, National Laboratory of Mineral Materials, School of Materials Science and Technology, China University of Geosciences, Xueyuan Road, Haidian District, Beijing 100083, China; 2003180010@cugb.edu.cn (Y.T.); rty5228@gmail.com (T.R.); 2103180007@cugb.edu.cn (L.Z.); 2103180008@cugb.edu.cn (J.Z.); 2 Jiaozuo Weina Technology Co., Ltd., Jiaozuo 454100, China; 15003916111@139.com; 3 Beijing Building Materials Academy of Sciences Research Co., Ltd., Shixing Street, Shijingshan District, Beijing 100041, China; ptliwei@163.com

**Keywords:** SiO_2_ microspheres, Na_2_SiO_3_, hydrolysis modification, nano-TiO_2_, composite photocatalyst

## Abstract

Modified microspheres (SiO_2_-M) were obtained by the hydrolytic modification of silicon dioxide (SiO_2_) microspheres with Na_2_SiO_3_, and then, SiO_2_-M was used as a carrier to prepare a composite photocatalyst (SiO_2_-M/TiO_2_) using the sol-gel method; i.e., nano-TiO_2_ was loaded on the surface of SiO_2_-M. The structure, morphology, and photocatalytic properties of SiO_2_-M/TiO_2_ were investigated. Besides, the mechanism of the effect of SiO_2_-M was also explored. The results show that the hydrolytic modification of Na_2_SiO_3_ coated the surface of SiO_2_ microspheres with an amorphous SiO_2_ shell layer and increased the quantity of hydroxyl groups. The photocatalytic performance of the composite photocatalyst was slightly better than that of pure nano-TiO_2_ and significantly better than that of the composite photocatalyst supported by unmodified SiO_2_. Thus, increasing the loading capacity of nano-TiO_2_, improving the dispersion of TiO_2,_ and increasing the active surface sites are essential factors for improving the functional efficiency of nano-TiO_2_. This work provides a new concept for the design of composite photocatalysts by optimizing the performance of the carrier.

## 1. Introduction

With social developments and the increasing scale of industrial production, water pollution, including the discharge of organic pollutants such as organic dyes and antibiotics, has become increasingly severe, posing a significant threat to human health and ecosystems [1,2]. At present, industries mainly purify wastewater by biodegradation, chemical precipitation, and adsorption, but generally suffer from high costs, low efficiency, and susceptibility to secondary pollution [3,4]. Inspired by photosynthesis, which uses light energy to drive chemical reactions, photocatalysis can degrade pollutants by using the strong oxidation of electron-hole pairs generated from semiconductors to purify wastewater and remediate the environment. Photocatalytic technology has become a research hotspot in the field of environmental purification [4,5,6] and has demonstrated broad application prospects.

Titanium dioxide nanoparticles (TiO_2_) are the photocatalytic materials most studied in recent years in preparation and applications, due to their non-toxicity, chemical stability, and superior performance [7,8]. However, TiO_2_ also suffers from an easy combination of photogenerated electrons and holes [9], low utilization of sunlight [10], reduced photocatalytic effect due to particle agglomeration [11], and difficult recycling in wastewater [12]. Elemental doping [13,14], noble metal deposition [15], heterostructure construction [11,14], and morphology control [16] have been adopted to improve TiO_2_ photocatalytic performance. It was found that loading TiO_2_ onto carriers enhances photocatalytic performance and enables the recycling and reuse of TiO_2_, resulting in lower application costs [17]. Krios et al. [18] strengthened the photocatalytic activity of TiO_2_ and enabled recycling owing to the effective interactions between TiO_2_ and zeolite by loading TiO_2_ onto the pretreated zeolite’s surface. Huang et al. [19] prepared TiO_2_/graphene composites using a solvothermal method. The interfacial chemical bonds in TiO_2_/graphene significantly reduced the recombination of electron-hole pairs and increased the quantity of holes involved in the photooxidation process. Zuo et al. [20] improved the photocatalytic efficiency of TiO_2_ by binding nano-TiO_2_ on the surface of diatomite with adsorption properties. Some studies synthesized core-shell structured TiO_2_ nanomaterials using noble metals as cores [21], and others prepared thin films by immobilizing TiO_2_ on solid support substrates (stainless steel, titanium alloys, titanium metal, and tin oxide-coated glasses) [22] to enhance TiO_2_ performance. However, the surface of theses carriers generally only has a small number of groups binding to TiO_2_, which is inconvenient for regulating the morphology of nano-TiO_2_. The uneven particle size of these carriers also reduces the performance of composite photocatalysts. Furthermore, solid support substrates, noble metals, and graphene as carriers are expensive, with a hydrophobic surface and difficult compatibility with the hybrid system. Therefore, it is of positive significance to select carriers with a homogeneous size, low price, and ability to improve the performance of loaded TiO_2_ nanoparticles through modification.

Silicon dioxide (SiO_2_) is commonly applied as a photocatalyst carrier, since it is rich in surface hydroxyl groups that can promote the dispersion and stability of the photocatalyst [23,24]. Many studies have confirmed the improved photocatalytic performance of composite material (SiO_2_/TiO_2_). Wang et al. [25] prepared TiO_2_-SiO_2_ core-shell nanocomposites with different coating thicknesses. The silica coating with a high specific surface area accelerated the removal of rhodamine B to some extent. Cizmar et al. [26] found that recombination between electrons and holes at the composite photocatalyst surface was reduced after copper modification, which improved the photocatalytic performance of the composite materials. SiO_2_ microspheres, a byproduct of the electrofusion process to produce zirconium oxide (ZrO_2_), mainly consist of spherical amorphous SiO_2_ particles. Such a byproduct can meet the requirements as a carrier to load TiO_2_ nanoparticles for its high purity, low price, high chemical, and high-temperature thermal stability [27,28]. SiO_2_ microspheres produced under high temperature and dry conditions have fewer surface hydroxyl groups, lower reactivity, and a smoother surface than that of SiO_2_ prepared in a liquid solution, resulting in its unsatisfying ability to be a substrate. Furthermore, through inorganic surface modification, the SiO_2_ surface can be coated with active ingredients to increase the number of surface hydroxyl groups, leading to the improvement of the surface activity of SiO_2_ microspheres. It is critical to keep the surface properties of silica under control because of the various silanol bases (≡Si-OH) reactions (such as chlorination, ammonification, and esterification) [29]. Wang et al. [17] treated SiO_2_ microspheres with sodium hydroxide solution to change the surface of microspheres from smooth to rough and increase the quantity of hydroxyl groups. It is important for improving the functionality of SiO_2_ microspheres loaded with nano-TiO_2_ and CdS photocatalysts. However, the modification by sodium hydroxide treatment conditions is harsh and corrosive to the equipment because of the reaction under a very high pH.

In this paper, the sodium silicate (Na_2_SiO_3_) hydrolysis method was used to modify SiO_2_ microspheres. Modified SiO_2_ (SiO_2_-M) was obtained by deposition of Na_2_SiO_3_ hydrolysate at the surface of SiO_2_ microspheres. Then, an SiO_2_-M/TiO_2_ composite photocatalyst was prepared through the sol-gel method. Furthermore, its structure, performance, and influence mechanism of SiO_2_-M were studied.

## 2. Experiment

### 2.1. Raw Materials and Reagents

The raw material of SiO_2_ microspheres was the byproduct of fused zirconia production, which was provided by Henan Jiaozuo Weina Powder Technology Co., Ltd., Jiazuo, China. It mainly comprised spherical particles with an amorphous SiO_2_ phase, and the main chemical composition (wt.%) was SiO_2_ 97.31 and ZrO_2_ 0.81. The test results show that the coarse end diameter (D90) was 4.973 μm, and the median diameter (D50) was 1.804 μm. The pure nano-TiO_2_ used in this study was prepared using the sol-gel method, and it was an anatase phase with a size of about 20–30 nm.

Tetra-butyl titanate ((C_16_H_36_O_4_)Ti) was used as a Ti^4+^ source and was a product of Beijing Yili Fine Chemicals Co., Ltd, Beijing, China. Acetyl acetone (C_5_H_8_O_2_) was a hydrolysis inhibitor, which was produced by Xilong Chemical Co., Ltd. (Guangzhou, China). Sodium silicate (Na_2_SiO_3_) from Beijing Yili Fine Chemicals Co., Ltd. was used as a modifier. Methyl orange was used as the target pollutant for photocatalytic degradation produced by Sinopharm Chemical Reagent Co., Ltd, Shanghai, China. Anhydrous ethanol (CH_3_CH_2_OH) deionized water was used as solvent.

### 2.2. Modification of SiO_2_ Microspheres

First, 2 g of SiO_2_ microspheres were decentralized in 100 mL of distilled water to form a dispersed suspension. Then, a certain amount of Na_2_SiO_3_ was put into the suspension to obtain a mixed solution. After stirring evenly, the mixed solution was moved to an oil bath at 80 °C for preheating for 20 min. In the meantime, 1 mol/L H_2_SO_4_ was added to the mixture dropwise to adjust the preset pH values. Then, the modified product was washed with deionized water five times after stirring for 3 h. Finally, the modified SiO_2_ microspheres (SiO_2_-M) were obtained after drying and grinding. For convenience of comparison, SiO_2_-M obtained at a different dosage of Na_2_SiO_3_ (0 g, 0.2 g, 0.4 g, 0.6 g, 1.0 g) and different pH values (pH = 9.0, 8.5, 8.0, 7.5, 7.0, 6.5) were denoted as X-SiO_2_-M-Y, where X represents the dosage of Na_2_SiO_3_ (g), and Y represents the pH value of solution during hydrolysis. The hydrolysis equation of Na_2_SiO_3_ was

SiO_3_^2−^ + 2H^+^ → SiO_2_↓ + H_2_O (1)

SiO_3_^2−^ + 2H^+^ + (n − 1)H_2_O → SiO_2_•nH_2_O↓ (2)

Thus, the surface modifiers of SiO_2_ microspheres in SiO_2_-M should be amorphous SiO_2_ and SiO_2_•nH_2_O.

### 2.3. Preparation of SiO_2_-M/TiO_2_ Composite Photocatalyst

First, 15 g of SiO_2_-M was put into a 500 mL conical flask with 140 mL of ethanol. After being evenly dispersed, 20 g of Tetra-Butyl Titanate (TBOT) was added dropwise to obtain a white suspension recorded as liquid A. Then, a mixture of 60 mL of anhydrous ethanol, 60 mL of deionized water, and 3 mL of acetylacetone was added to liquid A dropwise to obtain the mixed solution B. After stirring for a while, the mixed solution B was centrifuged to obtain the precipitate. The white sediments were washed with distilled water and dried to obtain the precursor of SiO_2_-M/TiO_2_. Finally, the precursor was calcined in a muffle furnace at 700 °C for 2 h to acquire composite photocatalysts.

### 2.4. Preparation of Pure TiO_2_

A total of 20 g of tetrabutyl titanate (TBOT) was added dropwise to 140 mL of anhydrous ethanol to obtain a yellow transparent solution. Then, a mixture of 60 mL of anhydrous ethanol, 60 mL of deionized water, and 3 mL of acetylacetone was added to the yellow transparent solution dropwise to form a mixed solution. Next, the TiO_2_ gel was obtained after stirring for a while. Finally, the gel was dried and calcined in a muffle furnace at 400 °C for 2 h to acquire pure nano-TiO_2_.

### 2.5. Testing and Characterization

Field emission scanning electron microscopy (SEM) (Gemini SEM, Carl Zeiss AG, Braunschweig, Germany) and high-resolution field emission transmission electron microscopy (TEM) (JEM-2100F, JEOL, Tokyo, Japan) were used to observe the morphology, structure, and size of SiO_2_ microspheres, and SiO_2_-M and SiO_2_-M/TiO_2_ particles. The elemental composition and content of the sample were measured by using a German Bruker S4-Explorer fluorescence spectrometer (XRF). The X-ray diffraction patterns were measured with a D8 advance X-ray diffractometer (Bruker, Karlsruhe, Germany) using Cu K_α_ radiation. The specific surface area of samples was tested by a nitrogen adsorption facility (Autosorb IQ, Quantachrome, Boynton Beach, FL, USA). The surface functional groups were examined with an infrared spectroscope (Spectrum 100, PerkinElmer, Shanghai, China) using KBr as background. The point of zero charge (PZC) was measured by using a Zetasizer Nano ZS90 (Malvern, Malvern City, UK). The amount of hydroxyl group on the surface of SiO_2_ microspheres was measured before and after the hydrolytic modification of Na_2_SiO_3_ by the acid–base titration method. Firstly, 2 g of the sample to be tested was added to 75 mL of NaCl solution (20 wt.%) and 25 mL of anhydrous ethanol to form a suspension. After the suspension was evenly stirred, the suspension’s pH was first reduced to 4 with 0.1 M HCl solution, and then, the pH of the suspension was increased to 9 by slowly adding a 0.1 M NaOH solution dropwise. The volume of NaOH solution used in the process was recorded, and the number of hydroxyl groups on the surface of the sample could be calculated using Equation (3).

N = 10^−18^(CVN_A_ × 10^−3^)/mS (3)

where N is the number of hydroxyl groups on the surface of the sample (/nm^2^), C is the concentration of NaOH solution (mol/L), V is the volume of NaOH solution used in the titration process (L), N_A_ is Avogadro constant (6.022 × 10^23^ mol^−1^), m is the sample mass (g), and S is the specific surface area of the sample (m^2^/g).

The degradation of methyl orange evaluated the photocatalytic performance of SiO_2_-M/TiO_2_ under ultraviolet light (the 300 W mercury lamp was used as the ultraviolet light source). The photocatalytic degradation tests involved in the experiments were carried out using a PhchemIII photoreactor from Beijing Newbit Technology Co., Ltd, Beijing, China. The reactor contains a light-source controller, stirrer, and circulation cooler, and can perform 12 sets of tests simultaneously. First, 50 mg SiO_2_-M/TiO_2_ was added to 50 mL of a certain concentration (C_0_) of methyl orange solution to obtain the mixture of the solid content of 1 g/L of suspension. Then, the suspension in dark conditions was stirred for 60 min to obtain SiO_2_-M/TiO_2_ on the adsorption equilibrium of methyl orange solution. After opening the ultraviolet light source mercury lamp (300 w), 5 mL of the sample was collected every 10 min. Next, the samples were centrifuged at 8000 r/min for 5 min to obtain the supernatant. Finally, the absorbance to the wavelength of 464 nm of the supernatant was measured by Cary 5000 UV-vis spectrophotometer (Agilent Technologies Inc, Santa Clara, CA, USA). The relationship between the absorbance and concentration was used to determine the concentration of methyl orange in the solution (C). The degradation effect of the photocatalyst was assessed by the change in C/C_0_. At the same time, the degradation extent D was obtained by C and C_0_, D = 100%(C_0_ − C)/C_0_.

## 3. Results and Discussion

### 3.1. Characterization of SiO_2_ Microspheres

#### 3.1.1. Morphology

**Figure 1:**
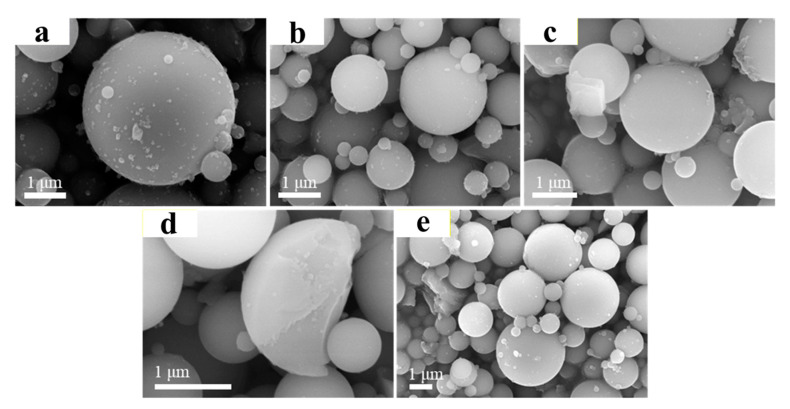
SEM images of (**a**) 0-SiO_2_-M, (**b**) 0.2-SiO_2_-M, (**c**) 0.4-SiO_2_-M, (**d**) 0.6-SiO_2_-M, and (**e**) 1.0-SiO_2_-M, respectively.

Figure 1 shows the SEM images of SiO_2_ microspheres modified with different Na_2_SiO_3_ dosages (the pH of hydrolysis was 8.5). As can be seen from this figure, the unmodified SiO_2_ microspheres (SiO_2_) are spherical with good dispersion. The diameter of the microspheres was about 0.5–2 μm, and the surface was smooth. However, a small amount of debris was attached to the unmodified SiO_2_ microspheres, which could be impurities in the raw material. Compared with the unmodified SiO_2_ microspheres, the spherical morphology of the modified SiO_2_-M remained unchanged, while the surface debris disappeared. It indicated that the hydrolytic modification with Na_2_SiO_3_ first led to the cleaning of impurities on the surface of the microspheres, which would reduce the interference of impurities and facilitate the loading of nano-TiO_2_ onto the surface of the SiO_2_ microspheres. By observing the surface of the fractured microspheres, it was found that a uniform shell coating formed on the surface of SiO_2_-M was amorphous SiO_2_ or SiO_2_•nH_2_O from the hydrolysate of Na_2_SiO_3_ (Figure 1d). As can be seen from Figure 1b–e, the hydrolysis products increased with the increase of Na_2_SiO_3_ dosage, so it can be presumed that the shell layer on the surface of SiO_2_ microspheres was continuously thickening and the coverage area increasing. As the hydrolyzed products of Na_2_SiO_3_ (SiO_2_ and SiO_2_•nH_2_O) were produced in solution, the quantity of hydroxyl groups on the microspheres’ surface was more than that on the surface of the unmodified microspheres. Therefore, it is speculated that the quantity of hydroxyl groups on the surface of the 0.6-SiO_2_-M microspheres was more than that on the surface of the unmodified microspheres, which undoubtedly becomes a critical factor in improving the level of nano-TiO_2_ supported and the properties of the composite photocatalyst. Besides, compared with 0.6-SiO_2_-M, the surface of 1.0-SiO_2_-M was smoother. Therefore, 0.6-SiO_2_-M was more suitable as a carrier.

**Figure 2:**
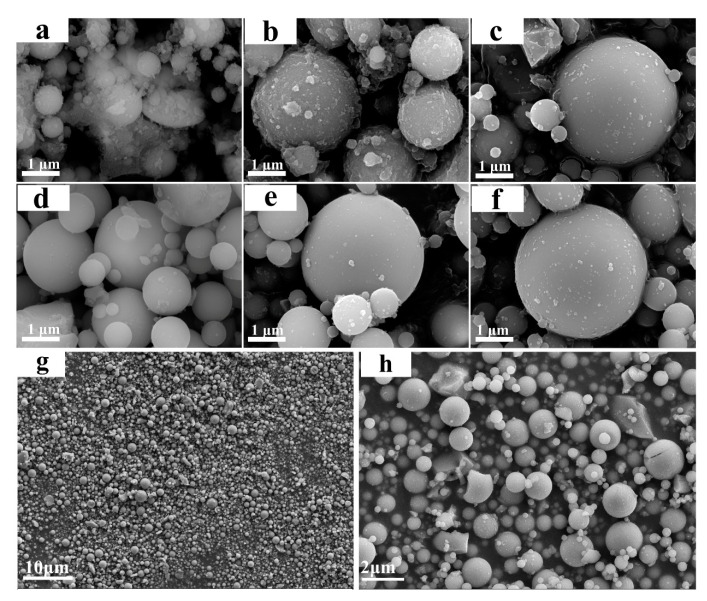
SEM images of (**a**) 0.6-SiO_2_-M-6.5, (**b**) 0.6-SiO_2_-M-7.0, (**c**) 0.6-SiO_2_-M-7.5, (**d**) 0.6-SiO_2_-M-8.0, (**e**) 0.6-SiO_2_-M-8.5, and (**f**) 0.6-SiO_2_-M-9.0, respectively; lower magnification SEM images of 0.6-SiO_2_-M-7.5 (**g**,**h**).

Figure 2 shows SEM images of a hydrolytic modification of SiO_2_ microspheres by Na_2_SiO_3_ (dosage 0.6 g) at a different pH of the suspension. A certain amount of acceptable debris can be seen on the whole surface of 0.6-SiO_2_-M-9 microspheres with uniform distribution. Due to the low H^+^ concentration in the system and weak hydrolysis reaction of Na_2_SiO_3_ when the pH value was 9, it is not enough to produce more hydrolysates to attach to the surface of SiO_2_ microspheres, nor could the impurity debris on the surface be cleaned off. As the pH value dropped to 8.5 and 8, H^+^ concentration and the hydrolysis degree of Na_2_SiO_3_ increased. Therefore, more hydrolysates were produced to attach to the surface of SiO_2_ microspheres, and impurities were removed, showing a gradually smooth surface (Figure 2d–f). When the pH value continued to drop to 7.5 and 7, the hydrolysis reaction of Na_2_SiO_3_ became more intense, and a large number of products could be produced in a short time. The excessive local concentration caused the hydrolysis products to nucleate spontaneously and created sedimentation on the surface of SiO_2_ microspheres in the form of particles, leading to a rough surface (Figure 2b,c). The pH value continued to decrease to 6.5, and the hydrolysis rate further accelerated. At this time, the hydrolysates rapidly nucleated, and a large number of particles formed, wrapping the surface of SiO_2_ microspheres (Figure 2a). These hydrolysates caused the agglomeration among SiO_2_ microspheres, which was not beneficial for loading nano-TiO_2_. According to the above results, Na_2_SiO_3_ hydrolytic modification was carried out when the pH value was 7.5. Figure 2g,h shows lower-magnification SEM images of 0.6-SiO_2_-M-7.5. It can be seen that the majority of the SiO_2_ originating from the hydrolysis of Na_2_SiO_3_ was not deposited separately from the surface of the microspheres, indicating that the microstructure of the sample was homogeneous.

Table 1 listed the quantity of hydroxyl groups on the surface of 0.6-SiO_2_-M-7.5 and unmodified SiO_2_ microspheres. Furthermore, 1.64 hydroxyl groups/nm^2^ for 0.6-SiO_2_-M-7.5 was 4.3 times higher than that of unmodified SiO_2_ microspheres (0.38 groups/nm^2^), indicating that the hydrolysis modification of Na_2_SiO_3_ substantially increased the quantity of hydroxyl groups on the surface of SiO_2_ microspheres. It became a critical factor in enhancing the carrier effect of SiO_2_ microspheres.

#### 3.1.2. Effect of Modified SiO_2_ Microspheres

**Figure 3:**
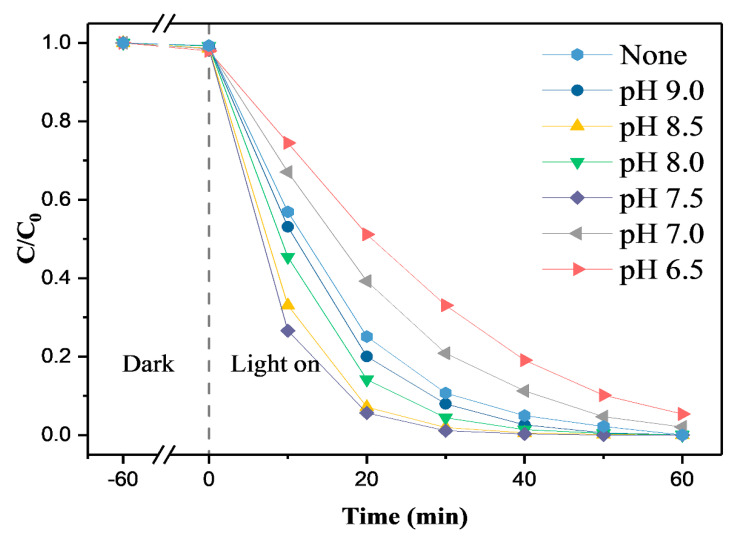
Effect of 0.6-SiO_2_-M-Y/TiO_2_ composite photocatalyst on the degradation of methyl orange.

To explore the effect of Na_2_SiO_3_, an SiO_2_-M/TiO_2_ composite photocatalyst was prepared via the sol-gel method using SiO_2_-M/TiO_2_ composite photocatalyst with Na_2_SiO_3_ content of 0.6 g and pH values of 9.0, 8.5, 8.0, and 7.5 as carriers. The degradation behavior of SiO_2_-M/TiO_2_ on methyl orange solution is shown in Figure 3. As can be seen from this figure, with the pH value of Na_2_SiO_3_ modification increasing from 6.5 to 7.5, the degradation efficiency of methyl orange by SiO_2_-M/TiO_2_ increased. The degradation efficiency was represented by a decrease in the C/C_0_ value of methyl orange solution at the same illumination time. With the continuous increase of the pH value to 9.0, the degradation efficiency of SiO_2_-M/TiO_2_ gradually decreased. By contrast, the degradation of SiO_2_-M/TiO_2_ was strongest at pH 7.5 and 8.5. The C/C_0_ values of MO with 0.6-SiO_2_-M-7.5/TiO_2_ and 0.6-SiO_2_-M-8.5/TiO_2_ were reduced to 0.01 and 0.02 after UV irradiation for 30 min, respectively. The degradation extent was 99% and 98%, respectively. The degradation extent reached 100% after 40 min of UV irradiation. It can also be seen from Figure 3 that the degradation extent of 0.6-SiO_2_-M-7.5/TiO_2_ and 0.6-SiO_2_-M-8.5/TiO_2_ was noticeably greater than that of the composite photocatalyst (SiO_2_/TiO_2_) with unmodified SiO_2_ microspheres as the carrier, indicating that the hydrolysis modification of Na_2_SiO_3_ significantly promotes the quality of nano-TiO_2_ supported by SiO_2_ microspheres.

Based on the results of SEM (Figure 2), it was also found that the photocatalytic effect of SiO_2_-M/TiO_2_ is closely related to the modification effect of SiO_2_ microspheres. According to Equations (1) and (2), low H^+^ concentration led to a decrease in hydrolysates, resulting in a decrease in the number of hydroxyl groups on the 0.6-SiO_2_-M-9.0 surface. It is detrimental to the loading of nano-TiO_2_, which reduced the photocatalytic performance of the samples. Large quantities of hydrolysates were formed due to the high H^+^ concentration, which results in the severe agglomeration of 0.6-SiO_2_-M-6.5. Therefore, the photocatalytic degradation ability of SiO_2_-M/TiO_2_ prepared by using 0.6-SiO_2_-M-6.5 as carriers was relatively weak. On the other hand, the surface of 0.6-SiO_2_-M-7.5 was coated with more hydrolysates with a large number of hydroxyl groups, while at the same time the dispersion of SiO_2_ microspheres was good. Thus, it exhibited a strong photocatalytic degradation effect.

#### 3.1.3. TGA and DSC of SiO_2_-M

**Figure 4:**
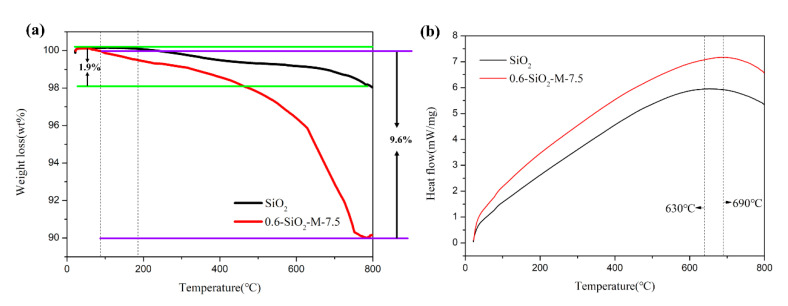
TGA curves of SiO_2_ and 0.6-SiO_2_-M-7.5 (**a**); DSC curves of SiO_2_ and 0.6-SiO_2_-M-7.5 (**b**).

To estimate the coating amount of SiO_2_-M, TGA-DSC curves of SiO_2_ and 0.6-SiO_2_-M-7.5 were measured. The results are shown in Figure 4. It can be seen that the whole TGA process of 0.6-SiO_2_-M-7.5 was divided into three stages: 25 °C to 80 °C, 80 °C to 180 °C, and 180 °C to 800 °C. The first stage was mainly caused by the evaporation of adsorbed water molecules and the second stage can be attributed to the removal of water from the SiO_2_•nH_2_O. The third stage was produced by condensation between adjacent hydroxyl groups [30]. In comparison, SiO_2_ had no significant weight loss until 180°C; this is because the raw material production of unmodified SiO_2_ microspheres was generated under high temperature solid phase conditions. The TGA curves suggested that the weight fraction of hydrolysate (amorphous SiO_2_ and SiO_2_•nH_2_O) in 0.6-SiO_2_-M-7.5 was approximately 7.7%.

### 3.2. Photocatalytic Performance

**Figure 5:**
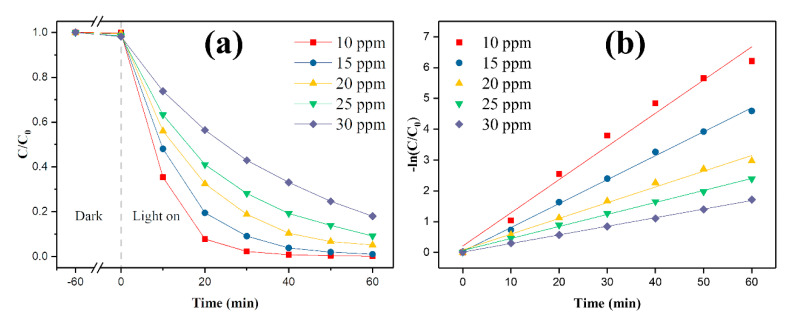
Effects of different initial concentrations on the degradation of methyl orange by 0.6-SiO_2_-M-7.5/TiO_2_ composite photocatalyst (**a**); degradation kinetics curve (**b**).

To investigate the effect of methyl orange concentration on degradation. The 1 g/L 0.6-SiO_2_-M-7.5/TiO_2_ was added to methyl orange solutions with different initial concentrations for UV light degradation, and the changes in C/C_0_ and −ln(C/C_0_) in methyl orange solutions after degradation are shown in Figure 5a,b, respectively. It is evident from Figure 5a that the degradation effect of 0.6-SiO_2_-M-7.5/TiO_2_ on the methyl orange solution of various original concentrations was gradually enhanced and finally reached a higher degradation extent with the increase of illumination time, indicating that it showed a strong degradation effect on the methyl orange solution of various concentrations. It was noted that the degradation extent of MO at different initial concentrations of 0.6-SiO_2_-M-7.5/TiO_2_ was significantly different. The degradation extent of 10 mg/L and 15 mg/L solution was the highest. When the illumination time was 40 min, the degradation extent reached 100% and 99%, respectively (C/C_0_ value was 0 and 0.01). As the initial concentration of methyl orange continued to increase to 20, 25, and 30 mg/L, the degradation rate of methyl orange gradually decreased, which was obviously caused by the high concentration of pollutants and the long reaction time. It can also be observed from Figure 5a that the extent of methyl orange degradation by 0.6-SiO_2_-M-7.5/TiO_2_ could still reach 80% (C/C_0_ was 0.2) when the illumination time was 60 min, indicating that a strong degradation effect could yet be achieved when the illumination time was adequately extended. The above results indicated that 0.6-SiO_2_-M-7.5/TiO_2_ could effectively degrade methyl orange solutions of different concentrations.

In addition, the degradation kinetics of MO by SiO_2_-M-/TiO_2_ were studied by using the Langmuir–Hinshelwood model to fit the experimental data. Due to the low concentration of reactants, the degradation process was considered to confirm the pseudo-first-order kinetic equation [31], as shown in Equation (3)

−ln(C/C_0_) = k_app_t (4)

where K_app_ is a pseudo-first-order degradation rate constant, reflecting the rate of degradation. It can be seen from Figure 5b that the R^2^ values of −ln(C/C_0_) -t fitting equation obtained by degradation of methyl orange at various initial concentrations were all greater than 0.98, indicating an excellent linear correlation. Thus, it conformed to the characteristics of a quasi-first-order reaction. Among them, K_app_ decreased with the growth of MO concentration, indicating the decrease of the degradation reaction rate, which was consistent with Figure 5a. Furthermore, when the initial concentration was 10 ppm, the photocatalyst degradation rate for pollutants decreased after 20 min of illumination. This was attributed to the fact that the pollutants in a lower initial concentration solution were mostly degraded after 20 min of light exposure, resulting in a decline in the number of degradable pollutants in the same amount of time. Therefore, the rate exhibited a decrease. Qin et al. [32] found that higher initial concentrations had higher photocatalytic true concentration ratios by listing the apparent reaction rate constants and the photocatalytic actual concentration ratios at different initial concentrations of pollutants, and higher initial concentrations could improve the efficiency of UV light utilization.

**Figure 6:**
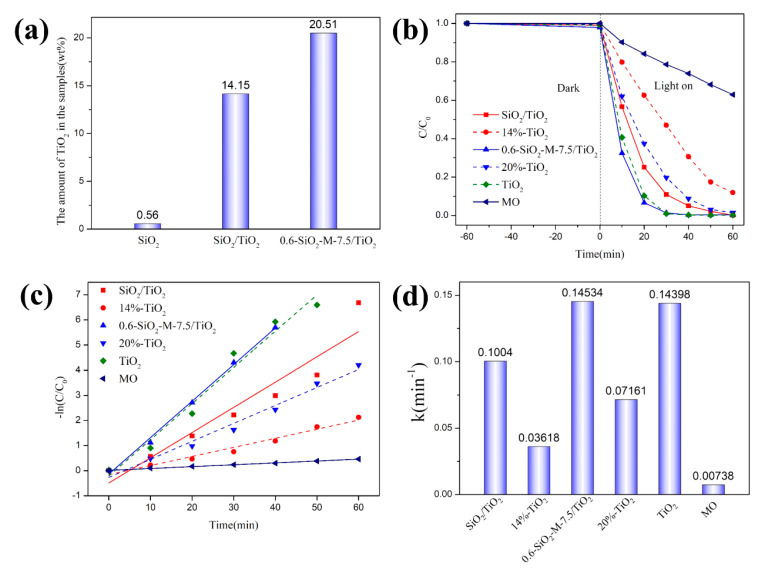
The amount of TiO_2_ in SiO_2_, SiO_2_/TiO_2_, and 0.6-SiO_2_-M-7.5/TiO_2_ (**a**); photocatalytic degradation of MO of the as-prepared composite as well as the comparative samples under ultraviolet light (**b**); photocatalytic degradation kinetics curve (**c**); apparent reaction rate constants (**d**).

To explore the effect of hydrolytic modification with Na_2_SiO_3_ on the photocatalytic performance of TiO_2_, the photocatalytic degradation behavior of 0.6-SiO_2_-M-7.5/TiO_2_ and its comparison samples was tested using methyl orange (MO) as the target pollutant (shown in Figure 6). Figure 6a shows the amount of TiO_2_ in SiO_2_, SiO_2_/TiO_2_, and 0.6-SiO_2_-M-7.5/TiO_2_. There was almost no TiO_2_ in SiO_2_ microspheres. In contrast, the amount of TiO_2_ in 0.6-SiO_2_-M-7.5/TiO_2_ was higher than that in SiO_2_/TiO_2_, indicating that the hydrolysis modification with Na_2_SiO_3_ increased the loading of nano-TiO_2_ onto the surface of SiO_2_. Figure 6b presents a comparison of the results for the degradation of MO (10 mg/L) by SiO_2_/TiO_2_, 0.6-SiO_2_-M-7.5/TiO_2_, and a physical mixture of TiO_2_ and SiO_2_-M (14%-TiO_2_: 14% TiO_2_ + 86% SiO_2_; 20%-TiO_2_: 20% TiO_2_ + 80% 0.6-SiO_2_-M-7.5; TiO_2_: 100% TiO_2_). It can be seen that SiO_2_/TiO_2_ and 0.6-SiO_2_-M-7.5/TiO_2_ exhibited a better degradation efficiency than 14%-TiO_2_ and 20%-TiO_2_, suggesting that both SiO_2_ and hydrolysis modification with Na_2_SiO_3_ have a certain enhancement on the photocatalytic reactivity of TiO_2_. Among all the samples, 0.6-SiO_2_-M-7.5/TiO_2_ showed an excellent degradation effect. The degradation extent of 0.6-SiO_2_-M-7.5/TiO_2_ was 93.4% under 20 min, and 100% under 40 min, which was much better than 20%-TiO_2_. The degradation rate constant of 0.6-SiO_2_-M-7.5/TiO_2_ was 0.14534 min^−1^, which was about 1.4, 4.0, and 2.0 times higher than that of SiO_2_/TiO_2_, 14%-TiO_2_, and 20%-TiO_2_, respectively (shown in Figure 6d). Besides, total organic carbon (TOC) was measured to evaluate the photocatalytic activity more accurately. The TOC content of methyl orange solution added with 0.6-SiO_2_-M-7.5/TiO_2_ was 15.8% of the original concentration, and that with nano-TiO_2_ was 15.1%. The degradation extent converted from the TOC was 84.2% and 84.9%, respectively. Methyl orange solution added with composite material and nano-TiO_2_ was nearly completely mineralized.

Although the degradation of methyl orange by SiO_2_-M-TiO_2_ composites was not significantly improved compared to that of nano-TiO_2_, considering that the proportion of nano-TiO_2_ in the composites was lower at the same catalyst dosage, the performance-enhancement effect due to loading was considered to be obvious. By using low-priced SiO_2_ microspheres as a carrier, the composite material was prepared by loading or coating nano-TiO_2_ particles with photocatalytic properties on the carrier surface, which not only reduced the cost by reducing the amount of nano-TiO_2_ particles but also maximized the function of nano-TiO_2_ particles for enhancing the utilization rate of TiO_2_. Furthermore, SiO_2_ microspheres as an inert carrier had a larger particle size than TiO_2_ on a macroscopic scale, making it possible to realize the recyclability of SiO_2_-M/TiO_2_ and further reduce the cost.

### 3.3. Morphology and Structure of Composite Photocatalyst

#### 3.3.1. Morphology

**Figure 7:**
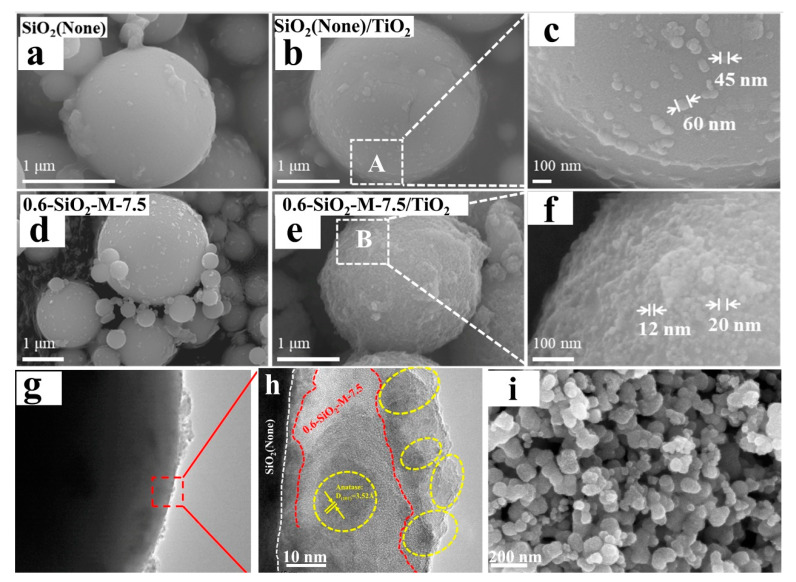
(**a**–**c**) SEM images of SiO_2_ and SiO_2_/TiO_2_; (**d–f**) SEM images of 0.6-SiO_2_-M-7.5 and 0.6-SiO_2_-M-7.5/TiO_2_; (**g**,**h**) TEM images of 0.6-SiO_2_-M-7.5/TiO_2_; (**i**) SEM of TiO_2_.

In order to understand the influence of Na_2_SiO_3_ hydrolytic modification on supported nano-TiO_2_, the morphologies of unmodified SiO_2_, 0.6-SiO_2_-M-7.5, SiO_2_/TiO_2,_ and 0.6-SiO_2_-M-7.5/TiO_2_ were characterized by SEM, and the results are shown in Figure 7a–f. As can be seen from Figure 6a,d, the surfaces of unmodified SiO_2_ and 0.6-SiO_2_-M-7.5 were relatively clean, and a small number of attached particles could be caused by impurities brought in by SiO_2_ raw materials and coating modification, respectively. By contrast, the surface morphology of SiO_2_/TiO_2_ and 0.6-SiO_2_-M-7.5/TiO_2_ showed a large number of attached particles, which was obviously caused by the loading of nano-TiO_2_. In particular, a large quantity of loadings on the surface of 0.6-SiO_2_-M-7.5/TiO_2_ basically formed a complete coverage of the SiO_2_ surface. As for SiO_2_/TiO_2_, loading TiO_2_ onto the surface created a smaller amount and only scattered on the surface of SiO_2_ microspheres (Figure 7b–f), suggesting that the hydrolysis modification of Na_2_SiO_3_ increased the loading of nano-TiO_2_ onto the surface of SiO_2_ microspheres. It is undoubtedly caused by the coverage of hydrolysis products around the SiO_2_-M surface, increased roughness, and the number of hydroxyl groups. It is consistent with the result that the photocatalytic degradation performance of 0.6-SiO_2_-M-7.5/TiO_2_ is better than that of SiO_2_/TiO_2_ (Figure 3). As seen from the SEM area enlargements of SiO_2_/TiO_2_ and 0.6-SiO_2_-M-7.5/TiO_2_ (Figure 7c,f), the particle sizes of TiO_2_ loaded onto the surfaces of both were 45–60 nm and 12–20 nm, respectively, and the latter was significantly smaller than the former.

The TEM image of 0.6-SiO_2_-M-7.5/TiO_2_ in Figure 7g shows that fine nano-TiO_2_ particles are evenly coated on the surface of SiO_2_ microspheres. It confirmed the results of SEM. Figure 7 shows an HRTEM image of the surface area of 0.6-SiO_2_-M-7.5/TiO_2_ particles at h, which could be divided into three parts. The black site was the part close to the surface of the SiO_2_ microsphere, with a relatively regular boundary. The red dotted line near the outer surface of the microsphere shows strong electron transmission and no lattice fringes. It is speculated that it is amorphous SiO_2_ or SiO_2_•nH_2_O hydrolysates of Na_2_SiO_3_. The outermost yellow dotted line is the crystalline phase with fringes. The measured fringe spacing was 0.35 nm, corresponding to the (101) surface spacing of anatase (ICDDcard#21-1272), indicating that this crystalline phase was supported by anatase-type nano-TiO_2_. Moreover, the grain size was about 10–20 nm, which is in agreement with the observation results of SEM. The results in Figure 7h show that the amorphous SiO_2_ or SiO_2_•nH_2_O coating layer was around the surface of SiO_2_ microspheres after hydrolytic modification by Na_2_SiO_3_, and then nano-TiO_2_ was supported on the surface of the coating layer to form a ternary composite structure.

**Figure 8:**
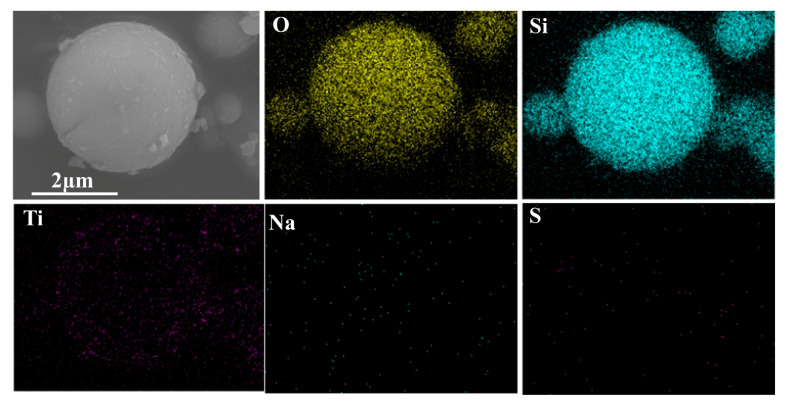
Element distribution of O, Si, Ti, Na, and S of 0.6-SiO_2_-M-7.5/TiO_2_.

Figure 8 shows the element mapping results of O, Si, Ti, Na, and S of 0.6-SiO_2_-M-7.5/TiO_2_. O and Si elements were uniformly distributed at the corresponding positions of 0.6-SiO_2_-M-7.5/TiO_2_ composite particles. Moreover, Ti elements were also uniformly distributed. It was further indicated that the small debris on 0.6-SiO_2_-M-7.5 could be nano-TiO_2_ particles.

#### 3.3.2. Crystalline and Structure

**Figure 9:**
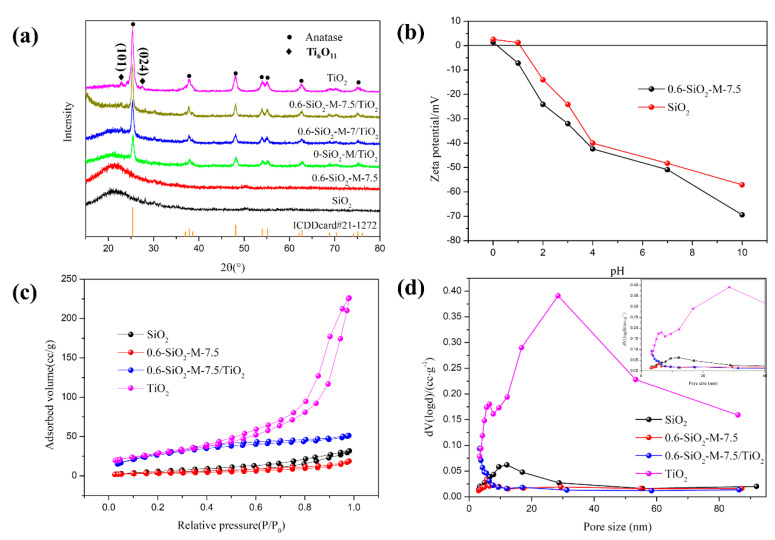
XRD patterns of SiO_2_, 0.6-SiO_2_-M-7.5, SiO_2_/TiO_2_, 0.6-SiO_2_-M-7/TiO_2_, 0.6-SiO_2_-M-7.5/TiO_2_, and TiO_2_ (**a**); relationship between zeta potential and pH (**b**); N_2_ adsorption–desorption curves; (**c**) and (**d**) pore-size distribution curves of SiO_2_, 0.6-SiO_2_-M-7.5, 0.6-SiO_2_-M-7.5/TiO_2_, and TiO_2_.

Figure 9a shows the XRD patterns of SiO_2_, 0.6-SiO_2_-M-7.5, SiO_2_/TiO_2_, 0.6-SiO_2_-M-7/TiO_2_, 0.6-SiO_2_-M-7.5/TiO_2_, and nano-TiO_2_. In the XRD pattern of SiO_2_, a weak and broad characteristic peak appeared between the diffraction angle (2θ) of 15 and 28°, indicating that the unmodified SiO_2_ had an amorphous structure. The XRD pattern of 0.6-SiO_2_-M-7.5 showed a stronger diffraction peak between 15 and 28° than SiO_2_, indicating that the modification of Na_2_SiO_3_ has no effect on the phase composition of SiO_2_. In the XRD patterns of the composite photocatalysts, diffraction peaks of anatase were present at 25.4°, 37.9°, 48.2°, 54.0°, 55.0°, 62.7°, and 75.1°, corresponding to the anatase (101), (004), (200), (105), (211), (204), and (215) crystal planes, respectively. The XRD patterns of 0.6-SiO_2_-M-7/TiO_2_ and 0.6-SiO_2_-M-7.5/TiO_2_ also exhibited only diffraction peaks of anatase compared to SiO_2_/TiO_2_, suggesting that different pH modifications did not affect the physical phase of nano-TiO_2_ in the composite samples. However, the characteristic diffraction peaks of anatase in both 0.6-SiO_2_-M-7/TiO_2_ and 0.6-SiO_2_-M-7.5/TiO_2_ were stronger than those of SiO_2_/TiO_2_, presumably due to the hydrolytic modification of Na_2_SiO_3_ to increase the amount of loaded TiO_2_ nanoparticles on the surface of SiO_2_. In addition, the XRD data were taken into the Scherrer formula, and the average size of the anatase phase TiO_2_ grains in SiO_2_/TiO_2_ and 0.6-SiO_2_-M-7.5/TiO_2_ was calculated to be 20 nm and 15 nm, respectively. It was hypothesized that there is less aggregation of TiO_2_ particles as a result of the formation of more Ti-O-Si bonds after the modification. Besides, in the XRD pattern of TiO_2_, diffraction peaks of Ti_6_O_11_ were present at 22.8° and 27.7°, corresponding to the anatase (101) and (024), respectively. This might be due to oxygen vacancy defects entering the TiO_2_ lattice, resulting in the formation of the Magnéli phase. Calcined at low temperatures, Ti_6_O_11_ fails to completely transform into anatase [33].

To investigate the change in electrical charges on the surface of SiO_2_ microspheres before and after modification, the points of zero charge (PZC) of 0.6-SiO_2_-M-7.5 and SiO_2_ were tested (Figure 9b). When the pH was lower than 1, the potential of 0.6-SiO_2_-M-7.5 (ζ_1_) was negative, indicating that the surface was negatively charged, while the surface of SiO_2_ was positively charged at this time (ζ_2_ was higher than 0). After the pH was greater than 1, the potentials of both became negative. However, the absolute value of ζ_1_ was consistently increased compared to the absolute value of ζ_2_, illustrating that the treatment modification of Na_2_SiO_3_ strengthened the negative charge on the surface of SiO_2_ microspheres. It was obviously due to the increase of the number of hydroxyl groups on the surface of SiO_2_ microspheres after modification, which was consistent with the results in Table 1. It also suggested that the modification was more favorable for the bonding between SiO_2_ and nano-TiO_2_.

The N_2_ adsorption-desorption isotherms and pore-size distribution of SiO_2_, 0.6-SiO_2_-M-7.5, 0.6-SiO_2_-M-7.5/TiO_2_, and nano-TiO_2_ are displayed in Table 2 and Figure 9c,d to explore the specific surface area and pore structure features. In Table 2, it can be noticed that SiO_2_ had a certain total pore volume, a small average pore size, and a mesoporous structure located at 10–15 nm (Figure 9d), which may be caused by the accumulation of microspheres. The decrease in specific surface area after modification was due to the coating of the modifier. In addition, the total pore volume and specific surface area of nano-TiO_2_ were significantly higher than the other samples and exhibited a broad pore distribution in the range of 20–50 nm (Figure 9d). This is reflected in the porous structural characteristics of the agglomerated TiO_2_ particle aggregates, which are in agreement with the SEM result of nano-TiO_2_ (Figure 7i). The total pore volume of the composite material was dramatically reduced, signaling the improved dispersion of TiO_2_ after loading. As seen in Figure 9c, the adsorption isotherms of nano-TiO_2_ belong to type IV of the adsorption isotherm. At pressures of P/P_0_ = 0.4 to 1.0, the adsorption volume increased substantially with increasing pressure. It suggested the presence of a certain amount of porous distribution apparently attributable to agglomeration. The adsorption isotherms of SiO_2_, 0.6-SiO_2_-M-7.5, and 0.6-SiO_2_-M-7.5/TiO_2_ revealed that they have almost no pore structure. By contrast, the unmodified SiO_2_ microspheres showed a moderate increase in adsorption with increasing pressure at high pressures of P/P_0_ = 0.8 to 1.0 compared with the modified microspheres, further confirming that the modification was beneficial in promoting the dispersibility of the microspheres.

### 3.4. Interaction of SiO_2_-M with Nano-TiO_2_

**Figure 10:**
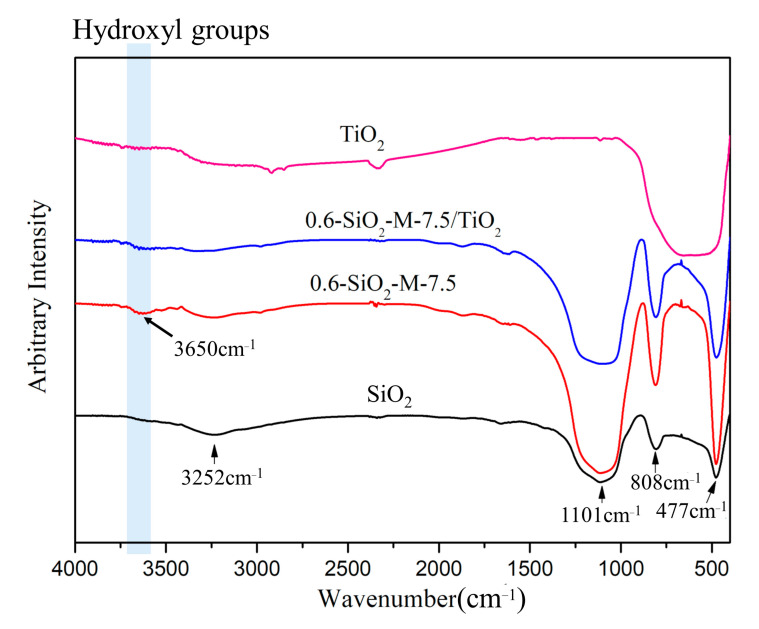
Infrared spectra of SiO_2_, 0.6-SiO_2_-M-7.5, 0.6-SiO_2_-M-7.5/TiO_2_, and TiO_2_.

To study the effect of modification on the surface properties of the material, the functional groups of SiO_2_, 0.6-SiO_2_-M-7.5, 0.6-SiO_2_-M-7.5/TiO_2_, and nano-TiO_2_ were tested by infrared spectroscopy (shown in Figure 10). The absorption peak at 1101 cm^−1^ in the SiO_2_ spectrum corresponds to the asymmetric stretching vibration of Si-O-Si [34]. The absorption peaks at 808 cm^−1^ and 477 cm^−1^ were attributed to the symmetric contraction and deformation vibrations of Si-O, and the absorption peak at 3252 cm^−1^ was the stretching and bending vibration of Si-OH or adsorbed water [35,36]. All of the above absorption peaks have been observed in the 0.6-SiO_2_-M-7.5 spectrum with an increase in peak intensity compared with SiO_2_. Moreover, a feature peak at 3650 cm^−1^ corresponded to the structural hydroxyl group, which was in agreement with the measured hydroxyl number results and supported the SEM and TEM results. In the 0.6-SiO_2_-M-7.5/TiO_2_ spectrum, the signature peak of the hydroxyl group was noticeably weaker. The absorption peaks at 1101 cm^−1^, 808 cm^−1^, and 477 cm^−1^ were also weaker and offset to a certain extent, but the peak’s intensity was stronger than SiO_2_. It suggested that nano-TiO_2_ and 0.6-SiO_2_-M-7.5 form a Ti-O-Si bond through the interaction of hydroxyl groups on the surface. The above results not only demonstrated that the modification increased the number of hydroxyl groups on the surface of SiO_2_ microspheres, but also suggested that TiO_2_ was stably coupled with the amorphous SiO_2_ and SiO_2_•nH_2_O by forming Ti-O-Si bonds.

### 3.5. Mechanism of SiO_2_-M to Enhance the Performance of Nano-TiO_2_

**Figure 11:**
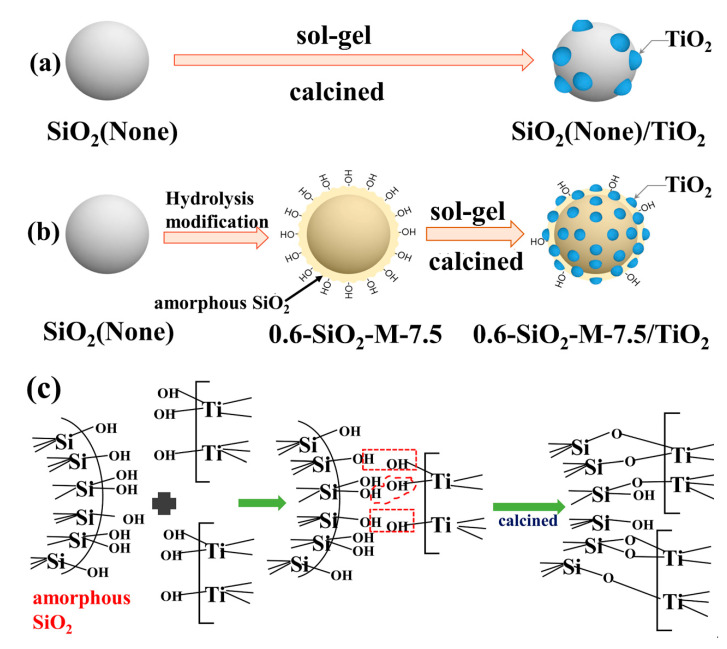
Schematic diagram of loading nano-TiO_2_ on SiO_2_ microspheres (**a**–**c**).

According to the above results, the mechanism of improvement of the photocatalytic performance of nano-TiO_2_ by SiO_2_-M supporting can be summarized. First, the raw material production of unmodified SiO_2_ microspheres was in the high-temperature solid-phase condition, resulting in extremely small amounts of hydroxyl groups on the surface. It was difficult for the colloidal particles of TiO_2_ precursors generated by the sol-gel method to combine with SiO_2_ microspheres through their respective surface hydroxyl groups bonding (forming Ti-OH---OH-Si and Ti-O-Si bonds). On the one hand, it led to loading only a small amount of nano-TiO_2_ on the surface of SiO_2_ microspheres. On the other hand, it led to an increased apparent granularity due to the agglomeration of the nano-TiO_2_. After the hydrolysis modification of SiO_2_ microspheres by Na_2_SiO_3_, the quantity of hydroxyl groups on the surface of the microspheres rose significantly due to the attachment of hydrolysate. Therefore, the loading amount of nano-TiO_2_ eventually increased. Second, in the preparation process of SiO_2_-M/TiO_2_, the increase of the quantity of hydroxyl groups on the surface of SiO_2_-M and the expansion of the surface binding with the nano-TiO_2_ precursor both reduced the size and dispersion of TiO_2_, leading to the rise of the exposed degree of the active spot. Thirdly, as the TiO_2_ scale loaded onto the surface of SiO_2_-M was reduced, it was beneficial to enhance the quantum effect of nano-TiO_2_. A schematic diagram reflecting the above mechanism and the preparation process of SiO_2_-M/TiO_2_ is shown in Figure 11.

## 4. Conclusions

By the hydrolytic modification of industrial by-product SiO_2_ microspheres with Na_2_SiO_3_, modified SiO_2_ microspheres (SiO_2_-M) coated with a certain amount of Na_2_SiO_3_ hydrolyzed products (amorphous SiO_2_ or SiO_2_•nH_2_O) were obtained. Compared with unmodified SiO_2_ microspheres, the quantity of hydroxyl groups on the surface of SiO_2_-M was significantly increased.

SiO_2_-M supporting nano-TiO_2_ composite photocatalyst (SiO_2_-M/TiO_2_) was prepared through the sol-gel method using SiO_2_-M as the carrier. SiO_2_-M/TiO_2_ had acceptable photocatalytic degradation of MO, comparable with that of pure nano-TiO_2_. The degradation performance of MO was noticeably better than that of unmodified SiO_2_ microspheres supporting nano-TiO_2_ products and slightly better than that of pure nano-TiO_2_. SiO_2_-M/TiO_2_ was formed by nano-TiO_2_ uniformly supported on the surface of SiO_2_-M; the size of TiO_2_ particles was 10–20 nm with good dispersion.

The influence mechanism of nano-TiO_2_ supported by SiO_2_-M to improve the photocatalytic performance and the hydrolysis modification of Na_2_SiO_3_ was as follows: The quantity of hydroxyl groups on the surface of SiO_2_-M increased, which enhanced the degree of binding with TiO_2_ precursors. As a result, the loading capacity of nano-TiO_2_ on the surface of SiO_2_-M was remarkably increased compared with that of unmodified SiO_2_ microspheres. The size of nano-TiO_2_ supported on the surface of SiO_2_-M was smaller than that on the surface of unmodified SiO_2_ microspheres, and the dispersion of nano-TiO_2_ was higher, which leads to increased exposure to the active site of nano-TiO_2_.

This study played a positive role in improving the property of supported nano-TiO_2_ and reducing the manufacturing and application photocatalyst cost by optimizing the carrier’s performance.

## Figures and Tables

**Table 1:** Number of surface hydroxyl groups of SiO_2_ microspheres before and after modification.

Sample	Number of Surface Hydroxyl Groups (/nm^2^)
SiO_2_ microspheres	0.38
0.6-SiO_2_-M-7.5	1.64

**Table 2:** Pore structure parameters of SiO_2_, 0.6-SiO_2_-M-7.5, 0.6-SiO_2_-M-7.5/TiO_2_, and TiO_2_ samples.

Sample	S_BET_/(m^2^/g)	V_Total_/(cm^3^/g)	D_Aver_/nm
SiO_2_	23.58	0.05	3.84
0.6-SiO_2_-M-7.5	10.49	0.03	5.61
0.6-SiO_2_-M-7.5/TiO_2_	21.12	0.04	3.39
TiO_2_	90.45	0.34	5.61

## Data Availability

Not applicable.
